# Recognizing Emotional Expression as an Outcome Measure After Face Transplant

**DOI:** 10.1001/jamanetworkopen.2019.19247

**Published:** 2020-01-15

**Authors:** Miguel I. Dorante, Branislav Kollar, Doha Obed, Valentin Haug, Sebastian Fischer, Bohdan Pomahac

**Affiliations:** 1 Division of Plastic Surgery, Department of Surgery, Brigham and Women’s Hospital, Harvard Medical School, Boston, Massachusetts; 2 Lahey Hospital and Medical Center, Department of Plastic and Reconstructive Surgery, Beth Israel Lahey Health, Burlington, Massachusetts; 3 Department of Hand, Plastic, and Reconstructive Surgery, Burn Trauma Center, BG Trauma Center Ludwigshafen, University of Heidelberg, Ludwigshafen, Germany

## Abstract

**Importance:**

Limited quantitative data exist on the restoration of nonverbal communication via facial emotional expression after face transplant. Objective and noninvasive methods for measuring outcomes and tracking rehabilitation after face transplant are lacking.

**Objective:**

To measure emotional expression as an indicator of functional outcomes and rehabilitation after face transplant via objective, noninvasive, and nonobtrusive software-based video analysis.

**Design, Setting, and Participants:**

This single-center case-control study analyzed videos with commercially available video analysis software capable of detecting emotional expression. The study participants were 6 patients who underwent face transplant at Brigham and Women’s Hospital between April 2009 and March 2014. They were matched by age, race/ethnicity, culture, and sex to 6 healthy controls with no prior facial surgical procedures. Participants were asked to perform either emotional expressions (direct evaluation) or standardized facial movements (indirect evaluation). Videos were obtained in a clinical setting, except for direct evaluation videos of 3 patients that were recorded at the patients’ residences. Data analysis was performed from June 2018 to November 2018.

**Main Outcomes and Measures:**

The possibility of detecting the emotional expressions of happiness, sadness, anger, fear, surprise, and disgust was evaluated using intensity score values between 0 and 1, representing expressions that are absent or fully present, respectively.

**Results:**

Six patients underwent face transplant (4 men; mean [SD] age, 42 [14] years). Four underwent full face transplants, and 2 underwent partial face transplants of the middle and lower two-thirds of the face. In healthy controls, happiness was the only emotion reliably recognized in both indirect (mean [SD] intensity score, 0.92 [0.05]) and direct (mean [SD] intensity score, 0.91 [0.04]) evaluation. Indirect evaluation showed that expression of happiness significantly improved 1 year after transplant (0.04 point per year; 95% CI, 0.02 to 0.06 point per year; *P* = .002). Expression of happiness was restored to a mean of 43% (range, 14% to 75%) of that of healthy controls after face transplant. The expression of sadness showed a significant change only during the first year after transplant (−0.53 point per year; 95% CI, −0.82 to −0.24 point per year; *P* = .005). All other emotions were detectable with no significant change after transplant. Nearly all emotions were detectable in long-term direct evaluation of 3 patients, with expression of happiness restored to a mean of 26% (range, 5% to 59%) of that of healthy controls.

**Conclusions and Relevance:**

Partial restoration of facial emotional expression is possible after face transplant. Video analysis software may provide useful clinical information and aid rehabilitation after face transplant.

## Key Points

**Question:**

Does face transplant restore the possibility of facial emotional expression, and can software-based video analysis be used to track progress over time?

**Findings:**

In this case-control study including 6 patients who underwent face transplant, all emotions were detectable, but only expression of happiness was reliably restored to 43% of the level of healthy controls and showed statistically significant improvement 1 year after transplant.

**Meaning:**

Software-based video analysis can be used as an objective, noninvasive, and nonobtrusive method of detecting and tracking facial emotional expression restoration after face transplant.

## Teaser

This case-control study uses software-based video analysis to detect and track restoration of facial emotional expression after face transplant.

## Introduction

Face transplant is a viable reconstructive option for patients with severe facial deformity that shows promising long-term results in improving functionality and quality of life.^1^ Outcome measures of face transplant have traditionally assessed the recovery of vital functions (eg, ability to breathe,^2^ eat, and speak^3^) and independent functions (eg, motor movement and protective and discriminative sensation),^4,5,6,7^ as well as the procedure’s functional psychological impact on quality of life and mental health.^8,9,10^ Measuring the restoration of these functions is necessary to determine the value of face transplant to the individual patient, but their recovery alone is not sufficient to achieve or explain societal reintegration after face transplant.

Nonverbal communication via facial emotional expression, a social function of the face, has evolved under the pressures of interacting in a social environment.^11^ Six specific emotional expressions—happiness, sadness, anger, surprise, fear, and disgust—are recognized across cultures and are the focus of social psychology research.^12,13,14^ Despite their high relevance, limited quantitative data are available on the restoration of facial emotional expression after face transplant. Existing evidence comes from methods such as facial surface electromyography,^15^ which is sensitive but requires painstaking placement of several electrodes on the skin,^16^ and appearance-based facial feature extraction, which is similar to facial recognition technology but requires significant data processing that limits reproducibility.^17^ These methods are obtrusive and prone to human instrumentation error. Their clinical implementation would be time-consuming and would bind patients to laboratory settings, which could affect medical adherence over time.^18^ The need to find a less obtrusive and more reliable method for evaluating emotional expression as an outcome measure of face transplant remains.

Software-based video analysis, a merger of facial recognition technology and deep learning, has proven to be capable of assessing facial motor movement functions after face transplant.^19^ We used a commercially available video analysis software that automatically analyzes facial movement for emotional expression to evaluate recovery of social functions after face transplant because it has been shown to do so in a manner similar to that of an objective human observer.^20^ Simultaneously, this method remains unobtrusive and capable of producing standardized measurements to track an individual’s rehabilitation progress or for group comparison.

We hypothesize that face transplant restores the possibility of emotional expression because it restores both the face’s human aesthetic and its underlying musculature, allowing for nonverbal communication perceivable to human observers. We believe that quantitative evaluation of emotional expression recovery in patients with face transplants could provide another objective outcome measure of face transplant. To our knowledge, this is the first study to detect and track facial emotional expression in patients with face transplants via an objective, noninvasive, and nonobtrusive method.

## Methods

### Study Participants

This study was approved by the institutional review board of the Partners Human Research Committee. Written informed consent was obtained from all study participants. This study follows the Strengthening the Reporting of Observational Studies in Epidemiology (STROBE) reporting guideline for case-control studies.

This retrospective case-control study was performed using 44 videos from 6 patients with face transplants (representing 15% of patients with face transplants worldwide) taken at regular intervals over a maximal posttransplant period of 9.5 years. Also used were 12 videos from 6 healthy controls who were matched according to the age of the donor and sex and cultural ethnicity of the recipient and who had no history of previous reconstructive or cosmetic facial procedures.

### Study Tools

Videos were acquired using a commercially available camera (EOS Rebel T3i; Canon) and tripod. Analysis was based on conventional video formats; thus, no special equipment or extra processing was required. FaceReader facial expression recognition software version 6.1 (Noldus) was used to detect and track faces, extract facial features, and analyze facial expressions.^21^ The software uses the Viola-Jones cascaded classifier algorithm^22^ to identify facial features and create a neutral face state. Then using the Active Appearance Method,^23^ an artificial face model is synthesized to compare vector variations between baseline and simulated faces with a database of annotated images.

**Figure 1.**
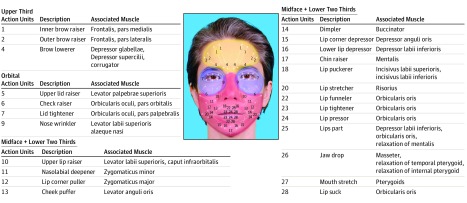
Action Units and Their Facial Regions The simple facial action units, as first described by Ekman et al,^12^ are presented with the muscles responsible for their motor movement. We assigned them into regions of the face commonly amenable to restoration by face transplant. Notably, there are no action units 3, 8, 19, and 21. Action units 25 and 26 are not represented visually because they are dependent on muscle relaxation. Action unit 27 is not represented visually because the force vector for the pterygoid muscles are not in the same plane of the image. Image by visual artist Coralie Vogelaar, 2018, used with permission.

The video analysis software achieves this by relying on the Facial Action Coding System,^24^ which taxonomizes visibly different facial movements on the basis of underlying anatomical structures into individual action units. For example, when people produce the prototypical facial expression for happiness, their cheeks raise (action unit 6) and the commissures of their mouth are pulled laterally (action unit 12). These facial expressions performed in concert effectively increase the width of the lower two-thirds of the face and may increase the distance from the chin to the brows, depending on the intensity of expression (Figure 1). The resultant smile is detectable and perceivable as the emotional expression for happiness, objectively, by untrained human observers^25^ and trained video analysis software.^26^

The video analysis software determines the magnitude of vector variation between neutral and simulated facial expressions using a trained artificial neural network^27^ and then compares them with prototypical features of 6 basic emotions to produce an intensity score for each. This intensity score value ranges from 0 to 1, depending on whether the emotional expression is entirely absent or fully present, respectively. According to the FaceReader software manufacturer, intensity score values greater than 0.5 are detectable by objective human observers.^21^

### Study Design

All study participants were recorded performing commands from 2 different protocols to either indirectly or directly evaluate emotional expression. For indirect evaluation, patients with face transplants were filmed every 6 months after transplant. All study participants performed a series of 12 facial movements: smile, frown, purse lips, open mouth wide, shut mouth tight, open eyes wide, close eyes tight, wrinkle nose, pucker lips, wink with right eye, wink with left eye, and puff cheeks. For direct evaluation, all healthy controls and 3 patients with face transplants (patient 1 at 9.5 years, patient 4 at 7.5 years, and patient 5 at 5.5 years) performed a series of 6 simulated faces for when they feel happy, sad, angry, surprised, scared, and disgusted. For both protocols, all study participants were asked to return to their neutral resting face between commands. Each video was less than 2 minutes long, and we attempted to standardize the background and lighting implemented.

Video analysis was performed after individual video calibration to correct for participant-specific biases toward certain facial expressions. Baseline emotional expressions were set to 0 (ie, intensity score equal to 0) using the first neutral resting face in each video. For indirect evaluation, maximum intensity score values for each emotion were used, and the possibility of expression was verified by correlating the protocol command, with correspondent action units, to the emotional state detected for consistency. For direct evaluation, the maximal intensity score values were extracted from the video sequence dedicated to the performed emotion. Data from all study participants were used for analysis of happiness. For all other emotions, patients with partial face transplants were excluded because not all action units necessary for the emotional expression were transplanted. For indirect evaluation, the highest intensity score value after the first year was chosen for each patient with face transplant to allow comparison with healthy controls.

### Statistical Analysis

To study changes in emotional expression over time in patients with face transplants, a piecewise linear regression model with random slope and intercept was fitted to the data with a knot at 1 year after transplant to allow for changes in the slope. The knot location was chosen because previous scientific literature^28,29^ reported that motor recovery improvements occurred mostly during the first year after transplant and because quantitative data beyond that time frame are mostly lacking. Statistical significance of the model was calculated from the comparison with hypothetical model, with a slope of 0. Two-sided *P* values less than .05 were considered statistically significant and were calculated using the exact sum-of-squares *F* test. The continuous parametric variables are presented as mean (95% CI) or mean (SD). All statistical analysis was performed using Prism statistical software version 8.02 (GraphPad Software). Data analysis was performed from June 2018 to November 2018.

## Results

### Study Participant Characteristics

Six patients underwent face transplant (4 men; mean [SD] age, 42 [14] years). Four patients received all action unit regions after full face transplant,^30,31,32,33^ whereas the 2 patients with partial face transplants received all and nearly all action units of the entire middle and lower two-thirds of the face (Table 1). The mean (SD) donor allograft age and mean healthy control age were both 48 (10) years. All patients underwent pertinent facial nerve and facial sensory nerve neurorrhaphies, bilaterally, with 3 patients requiring intraoperative nerve grafts, 1 of which required a revision nerve transfer (Table 1).

**Table 1.** Characteristics of Patients With Face Transplants and Healthy Controls

Patient No.	Date of Transplant	Age and Sex			Indication	Allograft Structures	Facial Nerve Neurorrhaphies	Sensory Neurorrhaphies	Nerve Transfers and Grafts		Transplanted Action Unit Regions
		Donor	Recipient	Healthy Controls					Intraoperative	Revision	
1	April 2009	Early 60s, male	Late 50s, male	Early 60s, male	High-voltage burn	Nose, cheeks, upper lip, midface bone^5^	5 Branches of the facial nerve, bilaterally^30^	Bilateral infraorbital and buccal nerves^30^	NA	NA	Midface plus lower two-thirds (except action unit 17)
2	March 2011	Late 40s, male	Mid 20s, male	Late 40s, male	High-voltage burn	Full face, partial scalp^31^	Only upper and lower divisions of the facial nerve on the left; frontal, zygomatic, buccal and marginal mandibular branches on the right^31^	Left mental nerve; right supraorbital, infraorbital, and mental nerves^31^	Recipient thoracodorsal nerve graft for superior and inferior divisions of facial nerve on the left, and marginal mandibular on the right^7^	NA	All
3	April 2011	Early 30s, male	Early 30s, male	Early 30s, male	High-voltage burn	Full face	Buccal and marginal mandibular branches of the facial nerve, bilaterally^31^	Bilateral supraorbital, infraorbital, and mental nerves^31^	NA	NA	All
4	May 2011	Early 40s, female	Late 50s, female	Late 40s, female	Animal attack	Full face, partial scalp, midface bone	6 Branches of the facial nerve, bilaterally, including frontal, zygomatic, buccal, and marginal mandibular^31^	Bilateral supraorbital, supratrochlear, and mental nerves^31^	Recipient great auricular nerve graft for 2 inferior buccal branches of facial nerve on the left^7^	11 mo after surgery; masseter to facial nerve transfer with recipient great auricular nerve interposition graft on the right^7^	All
5	February 2013	Mid 50s, female	Mid 40s, female	Early 50s, female	Chemical burn	Full face	5 Branches of the facial nerve, bilaterally^32^	Bilateral supraorbital, buccal, and mental nerves^32^	Nerve grafts had to be used on the left	NA	All
6	March 2014	Early 50s, male	Late 30s, male	Late 40s, male	Self-inflicted gunshot wound	Nose, lower two-thirds, maxilla, mandible	Buccal and marginal mandibular branches of the facial nerve, bilaterally^33^	Infraorbital nerves, bilaterally^33^	NA	NA	Midface plus lower two-thirds

Abbreviation: NA, not applicable.

### Evaluation of Healthy Controls

The healthy control videos were analyzed first to validate the sensitivity and specificity of both protocols and the video analysis software. Only the emotion of happiness could be reliably detected, with mean (SD) intensity score values of 0.92 (0.05) for indirect evaluation and 0.91 (0.04) for direct evaluation (Table 2 and eFigure 1 in the Supplement). All other emotions were detectable, but mean intensity score values did not pass the threshold for objective observer detection during both indirect and direct evaluation (eFigure 1 in the Supplement).

**Table 2.** Evaluation of Emotional Expression^a^

Emotional Expression	Action Units Involved	Patients With Face Transplant, Mean (SD), Intensity Score Value		Healthy Controls, Mean (SD), Intensity Score Value	
		Indirect Evaluation (n = 6)	Direct Evaluation (n = 3)	Indirect Evaluation (n = 6)	Direct Evaluation (n = 6)
Happiness	6 + 12	0.38 (0.24)	0.24 (0.26)	0.92 (0.05)	0.91 (0.04)
Sadness	1 + 4 + 15	0.34 (0.16)	0.13 (0.11)	0.44 (0.28)	0.34 (0.31)
Anger	1 + 2 + 5 + 26	0.17 (0.21)	0	0.33 (0.16)	0.28 (0.21)
Surprise	1 + 2 + 4 + 5 + 7 + 20 + 26	0.28 (0.23)	0.22 (0.37)	0.47 (0.14)	0.19 (0.14)
Fear	4 + 5 + 7 + 23	0.24 (0.16)	0.06 (0.09)	0.23 (0.16)	0.11 (0.15)
Disgust	9 + 15 + 16	0.09 (0.10)	0.02 (0.02)	0.34 (0.26)	0.19 (0.25)

^a^ The results for the indirect and direct evaluation of emotional expression in patients with face transplants and healthy controls are displayed. For each emotion, their corresponding action units are included and results display the mean (SD) intensity score values. For indirect evaluation, the highest intensity score value after the first year was chosen for each patient with a face transplant to allow comparison with healthy controls.

### Indirect Evaluation of Patients With Face Transplant

**Figure 2.**
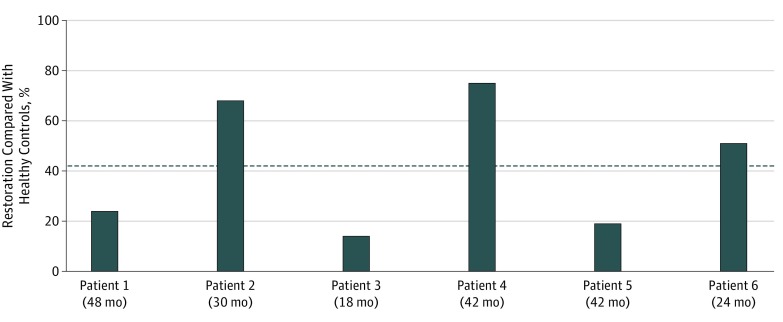
Restoration of Happiness Expression After Face Transplant Compared With Healthy Controls For indirect evaluation of expression of happiness, the maximum intensity score values of each patient with face transplant after the first posttransplant year were compared with the mean intensity score values of healthy controls. We found that expression of happiness, based on the recovery of the ability to smile (action units 6 + 12), was restored to a mean of 43% of that of healthy controls (dashed line) in the first 5 years after transplant.

We found that the emotional expression of happiness, sadness, anger, surprise, fear, and disgust was possible after face transplant with nonzero intensity score values detectable in all patients with face transplants. The mean (SD) group intensity score values were 0.38 (0.24) for happiness, 0.34 (0.16) for sadness, 0.17 (0.21) for anger, 0.28 (0.23) for surprise, 0.24 (0.16) for fear, and 0.09 (0.10) for disgust (Table 2). To determine percentage recovery, maximum intensity score values for each patient were compared with mean intensity score values of healthy controls for every emotion. This made recovery greater than 100% of the level of healthy controls possible for individual patients with face transplants. Happiness expression after face transplant was found to be restored to 43% (range, 14%-75%) of that of healthy controls (Figure 2), with other emotions compared in similar fashion but yielding unreliable results (eFigure 2 in the Supplement).

**Figure 3.**
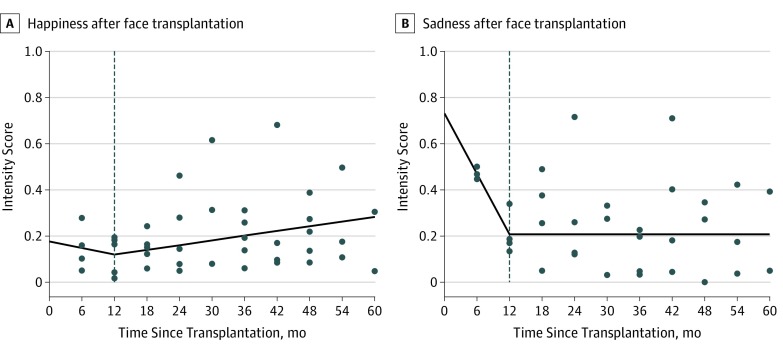
Longitudinal Evaluation of Expression of Happiness and Sadness After Face Transplant The intensity score values for expression of happiness and sadness during longitudinal indirect evaluation were modeled using piecewise linear regression with a knot at posttransplant year 1 (dashed lines). A, Expression of happiness was found to increase significantly by 0.04 point per year (95% CI, 0.02 to 0.06 point per year; *P* = .002) after the knot at year 1 (before year 1, −0.06 point per year; 95% CI, −0.34 to 0.23 point per year). B, The intensity score values for expression of sadness decreased significantly by 0.53 point per year in posttransplant year 1 (95% CI, −0.82 to −0.24 point per year; *P* = .005), but afterward the change was negligible (0.01 point per year, 95% CI, −0.01 to 0.03 point per year; *P* = .48).

Intensity score values for indirect evaluation of patients with face transplants were tracked over time. During posttransplant year 1, the intensity score values for happiness decreased nonsignificantly by 0.06 point per year (95% CI, −0.34 to 0.23 point per year; *P* = .66). Afterward, intensity score values for happiness increased significantly by 0.04 point per year (95% CI, 0.02 to 0.06 point per year; *P* = .002) (Figure 3). The intensity score values for sadness decreased significantly by 0.53 point during posttransplant year 1 (95% CI, −0.82 to −0.24 point per year; *P* = .005), with negligible changes afterward (0.01 point per year; 95% CI, −0.01 to 0.03 point per year; *P* = .48) (Figure 3). Individual patient trends for happiness and sadness are displayed in eFigure 3 in the Supplement. The remaining emotions of anger, surprise, fear, and disgust had intensity score values with nonsignificant changes (*P* > .05) after transplant (eFigure 4 in the Supplement).

### Long-term Direct Evaluation of Patients With Face Transplant

Three of 6 patients with face transplants were able to be filmed for the direct evaluation protocol. The mean (SD) group intensity score values were 0.24 (0.26) for happiness, 0.13 (0.11) for sadness, 0 for anger, 0.22 (0.37) for surprise, 0.06 (0.09) for fear, and 0.02 (0.02) for disgust (Table 2). On the basis of limited cohort data, expression of happiness was restored to a mean of 26% (range, 5%-59%) of that of healthy controls in the long term (eFigure 5 in the Supplement).

## Discussion

Facial recognition technology has been proposed as a method to improve performance metrics in vascularized composite allotransplant.^34^ In this study, we show that using software-based video analysis to detect and track nonverbal communication via facial emotional expression after face transplant is feasible. This method is noninvasive, nonobtrusive, able to be implemented widely, and capable of producing objective intensity score values that are amenable to standardization. All 6 basic emotions—happiness, sadness, anger, surprise, fear, and disgust—were detectable with nonzero intensity score values in patients with face transplants during indirect evaluation. This finding suggests that restoration of functional human aesthetic and underlying facial musculature after face transplant surpasses the threshold necessary for objective perception by software.

A prior study^19^ using software-based analysis showed that smile significantly improves over time after face transplant and is comparable with that of the healthy population, consistent with reported outcomes from most other face transplant teams.^5^ The current study found that the potential to express happiness after face transplant recovers to 43% of that of healthy controls in the first 5 years; after year 1, expression of happiness significantly improves by 0.04 intensity score point per year. These findings are significant because they are the first objective values on smile restoration after face transplant that can be standardized and tracked over time. Despite having a mean intensity score value less than 0.50, or below the threshold for recognition by objective human observers, happiness may be the only valid and reliable marker after face transplant. This emotional expression is uniquely and reliably recognized in healthy controls with high specificity and sensitivity. Furthermore, given that the video analysis software recognizes happiness similarly to objective human observers with accuracy greater than 90%,^25,35,36,37^ its ability to detect happiness after face transplant at subclinical levels not perceptible to human observers highlights the potential of video analysis software as a rehabilitative tool.

Although reliable interpretation is possible for happiness only, sadness was the only other expression that significantly changed after transplant. We believe that the high intensity score values before year 1 are the result of tissues drooping because of their weight and from incomplete recovery of muscle tone. Increased intensity score values for sadness in all patients with face transplants before calibration before year 1 further support this theory. Given no significant change in sadness expression thereafter, the theory that neuromuscular recovery after year 1 creates a true baseline expression of the new face is supported.

Both the present study and our previous study^19^ using facial recognition technology independently validate our findings that mean motor function at 5 years after transplant reaches 60% of maximal possible recovery.^1^ Currently, we evaluate motor recovery using the Daniels and Worthingham manual muscle testing technique,^38^ which is the standard for measurement but is liable to subjective evaluation, time-consuming, and exhausting for patients. Using software-based video analysis to measure underlying muscle recovery indirectly via emotional expression could address these limitations and provide novel information about a patient’s recovery. In essence, this tool measures the return of recognizable human aesthetic and nonverbal communication, both social functions of the face, in an unbiased manner. It can objectively provide data helpful to determine whether face transplant meets its ethical goal of restoring functions necessary for societal reintegration.

Some studies have attempted to measure emotional expression after face transplant. Topçu et al^15^ found that the frequency and spatial distribution of facial surface electromyography data recorded during emotional expression were significantly different between patients with face transplants and healthy controls after 2 years. One of their 3 patients with full face transplants had high-frequency firing distribution similar to that of healthy controls for happiness, whereas other expressions had similar distribution patterns but lower frequency. This could be due to limitations in electromyography measurement of emotional expression.^39^ Supporting this claim are findings from De Letter et al^40^ showing that electromyography detected signs of remyelinization after face transplant without clinically meaningful return of function. Bedeloglu et al^17^ acknowledged this limitation, performed image-based analysis of emotional expression in 2 patients with full face transplants, and found that happiness could be detected to 45% of that of healthy controls after 3 years. Their methods, despite being similar to processes required for software analysis and yielding results comparable to ours, require trained interpretation of the data. The video analysis software in our study has sensitivity on par with that of electromyography,^41^ but because it depends on visual data necessary for human visual system processing,^26,42^ clinical specificity for emotional expression detection is greater. Reliability of the tool improves with optimization of lighting and background, and when it is combined with easily interpretable intensity score values amenable to standardization and reduced sensitivity to human instrumentation error, the argument for video analysis software as a clinical assessment tool is stronger than that for electromyography.

Future research should prioritize face transplant rehabilitation programs. Topçu et al^43^ observed gradual recovery of emotional expressions after rehabilitation with functional electrical stimulation in 2 patients with full face transplants after 3 years. Their findings are subject to the same pitfalls of using electromyography and would make long-term comparison unreliable given the high likelihood of human instrumentation error. Incorporating software-based video analysis into posttransplant rehabilitation could allow for both intensity score value tracking and personalized rehabilitation goals based on expected face transplant cohort data. A second research focus should seek to improve on the Cleveland Clinic FACES Scoring System for Face Transplant Candidate Evaluation.^44,45^ Development of a specific functional outcome scale for face transplant should rely on objective measures of vital, independent, psychological, and social functions of the face. Software-based video analysis could provide objective intensity score values to standardize a grading system for the recovery of emotional expression potential after face transplant. Another area for implementing software-based video analysis is the detection of allograft rejection. Visible redness and swelling of the facial allograft are associated with rejection episodes within the first 2 years.^46^ These may be features amenable to software detection that could provide objective data to supplement more-specific biomarkers of rejection.^47^

### Limitations

Motivation of patients to perform facial expressions and their comfort in expressing emotions is a limitation of this study. Rehabilitation after face transplant is strenuous because it burdens the patient with numerous, lengthy appointments that can result in follow-up fatigue. This could affect participation in research and may be responsible for intensity score variability within the same patient and between patients. Software-based video analysis could address follow-up fatigue by reducing the need for long-distance travel to the transplant hospital.^48^ Videos could be filmed at a patient’s home, analyzed, and then shared with the transplant team, as was done for long-term direct evaluation in patients with face transplants for this study. This could facilitate a more honest appreciation of functional recovery outside of a laboratory setting,^49^ aid rehabilitation,^50^ and, on the basis of solid-organ transplantation data, may improve medical adherence and outcomes while decreasing the economic cost after transplant.^51^

Objective evaluation of emotional expressions after face transplant is a challenge because of cultural, regional,^52^ and personal variance in expression and presents another limitation to this study. The deep learning algorithm in the video analysis software is trained on images displaying prototypical emotions specific to Western cultures, with exaggerated facial expressions that may not depict realistic expression by individuals. It is possible that not every study participant performed all prototypical features while making facial emotional expressions under direct evaluation. This could explain why intensity score values for healthy controls under direct evaluation demonstrated large variability due to personal differences in expressivity. These biases could also explain why only happiness was reliably recognized in healthy controls, because its recognition is dependent on a smile that requires 2 action units to perform. More-complex emotional expressions, such as fear, require a greater number of units firing in concert to be detected, allowing for greater likelihood of personal and cultural biases to affect individual emotional expression.

Along these lines, new research shows that Eastern and Western cultures appreciate facial emotional expressions differently,^53,54,55^ which suggests that universality in expression relies more on valence, arousal, and dominance.^56,57^ We believe our findings support this latter point in 2 ways. Happiness and sadness (positive and negative valence, respectively) were the only emotions that were detected with statistical significance and were the 2 emotions with the largest mean intensity score values under direct evaluation in healthy controls. Interestingly, for all study participants, the mean intensity score values were greater for indirect evaluation of every emotional expression than for direct evaluation while maintaining similar variability. This could be explained by decreased effort and recruitment of motor units necessary to activate single action units. However, this would only explain these findings for simple emotional expressions, such as happiness and sadness, but not for more complex emotional expressions, such as anger, surprise, fear, and disgust, which require multiple action units firing simultaneously to be detected. This suggests that action units necessary for emotional expression detection are functional after face transplant and cultural bias in emotional expression was mitigated under indirect evaluation.

There is evidence that face transplant restores the potential to perform a function important for social integration,^58^ one that provides clues on biological and mental health.^59,60^ Although our results are reflective of only 15% of patients with face transplants worldwide, future collaborative studies between face transplantation centers should validate our results. If and when face transplantation occurs in Eastern societies, software will have to be trained on a more culturally appropriate set of images to accurately detect emotional expression as an outcome.

## Conclusions

This study demonstrates the potential for quantitative evaluation of emotional expression after face transplant to complement existing outcome measures. We believe that vital and independent functions restore the hardware of the face, with psychological and social functions acting as its necessary software. The use of video analysis software—an objective, noninvasive, and nonobtrusive method—to detect and track emotional expression in patients with face transplants provides novel evidence supporting the procedure’s viability as a treatment modality for severe facial deformity. Results from our case-control study suggest that partial restoration of facial emotional expression is possible after face transplant. The results indicate the potential for video analysis software to provide useful clinical information and aid rehabilitation after face transplant.

## Notes

Supplement. **eFigure 1. **Emotional Expression in Healthy Controls **eFigure 2. **Comparison of Emotions **eFigure 3. **Individual Patient Trends of Longitudinal Evaluation of Happiness and Sadness After Face Transplantation **eFigure 4. **Longitudinal Evaluation of Emotions After Face Transplantation **eFigure 5. **Long-term Comparison of Happiness
