# Supplementary material for: Recognizing Emotional Expression as an Outcome Measure After Face Transplant
Source: JAMA Netw Open. 2020 Jan 15;3(1):e1919247. doi: 10.1001/jamanetworkopen.2019.19247 (PMC6991259; doi:10.1001/jamanetworkopen.2019.19247)
Supplement: Supplement. — eFigure 1. Emotional Expression in Healthy Controls eFigure 2. Comparison of Emotions eFigure 3. Individual Patient Trends of Longitudinal Evaluation of Happiness and Sadness After Face Transplantation eFigure 4. Longitudinal Evaluation of Emotions After Face Transplantation eFigure 5. Long-term Comparison of Happiness [file jamanetwopen-3-e1919247-s001.pdf]

Supplementary Online Content

Dorante MI, Kollar B, Obed D, Haug V, Fischer S, Pomahac B. Recognizing emotional expression as an outcome measure after face transplant. *JAMA Netw Open*. 2020;3(1):e1919247. doi:10.1001/jamanetworkopen.2019.19247

eFigure 1. Emotional Expression in Healthy Controls
eFigure 2. Comparison of Emotions
eFigure 3. Individual Patient Trends of Longitudinal Evaluation of Happiness and Sadness After Face Transplantation
eFigure 4. Longitudinal Evaluation of Emotions After Face Transplantation
eFigure 5. Long-term Comparison of Happiness

This supplementary material has been provided by the authors to give readers additional information about their work.

© 2020 Dorante MI et al. *JAMA Network Open.*

eFigure 1. Emotional Expression in Healthy Controls

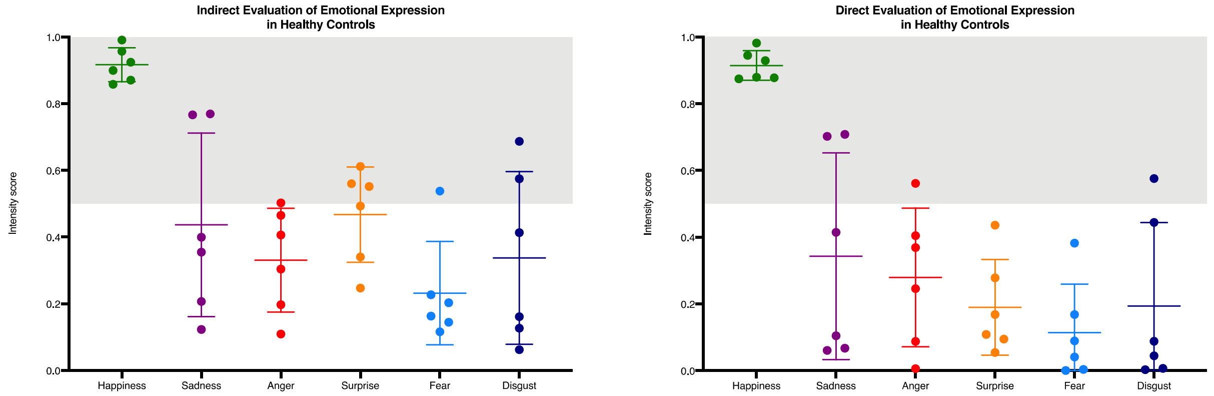

Side-by-side comparison of indirect and direct evaluation of emotional expressions in healthy controls. The emotion of happiness was detected with the least variability and with average intensity score values above the threshold for detection (grey area) by objective human observers in both protocols. Data is presented as scatter dot plot showing every individual value. Mean and standard deviation are displayed as long and short horizontal lines, respectively.

© 2020 Dorante MI et al. *JAMA Network Open.*

eFigure 2. Comparison of Emotions

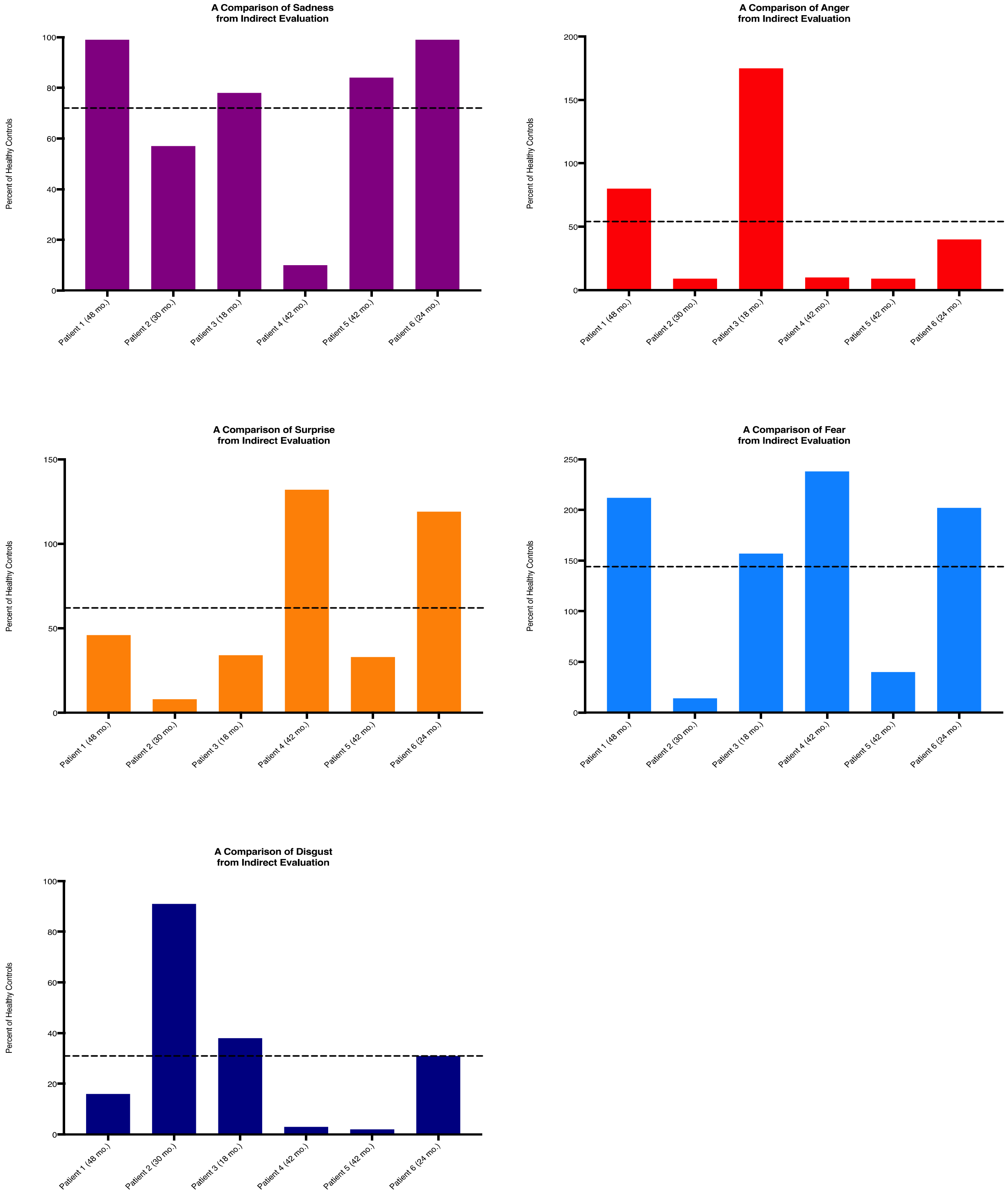

For the indirect evaluation of sadness, anger, surprise, fear and disgust, the maximum intensity score values of each patient with a face transplant after first post-transplant year were compared to the average intensity score value of healthy controls. The dotted horizontal line in each graph represents the mean restoration of each emotion after face transplantation. We found that: sadness (Action Units 1+4+15) is restored to a mean of 72% of healthy subjects; anger (Action Units 1+2+5+26) is restored to a mean of 80% of healthy subjects; surprise (Action Units 1+2+4+5+7+20+26) is restored to a mean of 62% of healthy subjects; fear (Action Units 4+5+7+23) is restored to a mean of 144% of healthy subjects; and, disgust (Action Units 9+15+16) is restored to a mean of 31% of healthy subjects.

© 2020 Dorante MI et al. *JAMA Network Open.*

eFigure 3. Individual Patient Trends of Longitudinal Evaluation of Happiness and Sadness After Face Transplantation

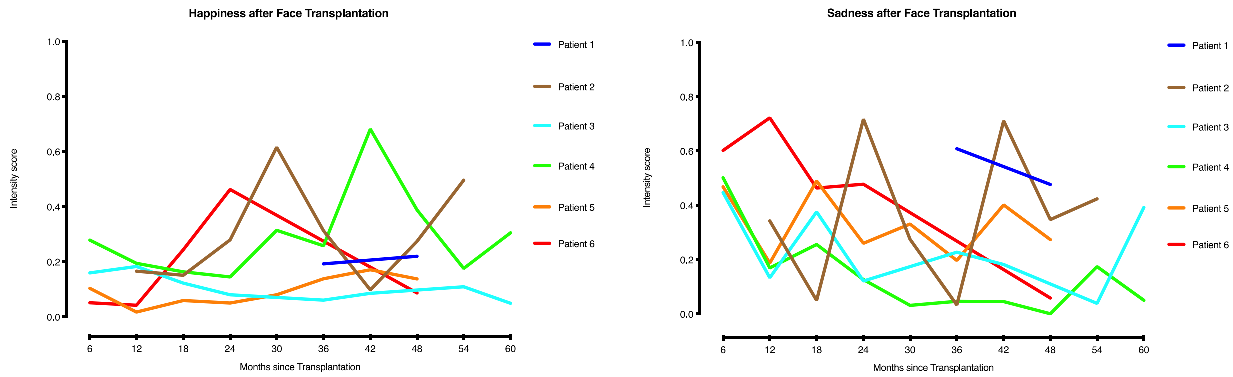

Individual patient trends that display the change in maximal intensity score values for happiness and sadness during longitudinal indirect evaluation. These trends follow the maximum intensity score values of happiness and sadness per patient per time point.

© 2020 Dorante MI et al. *JAMA Network Open.*

eFigure 4. Longitudinal Evaluation of Emotions After Face Transplantation

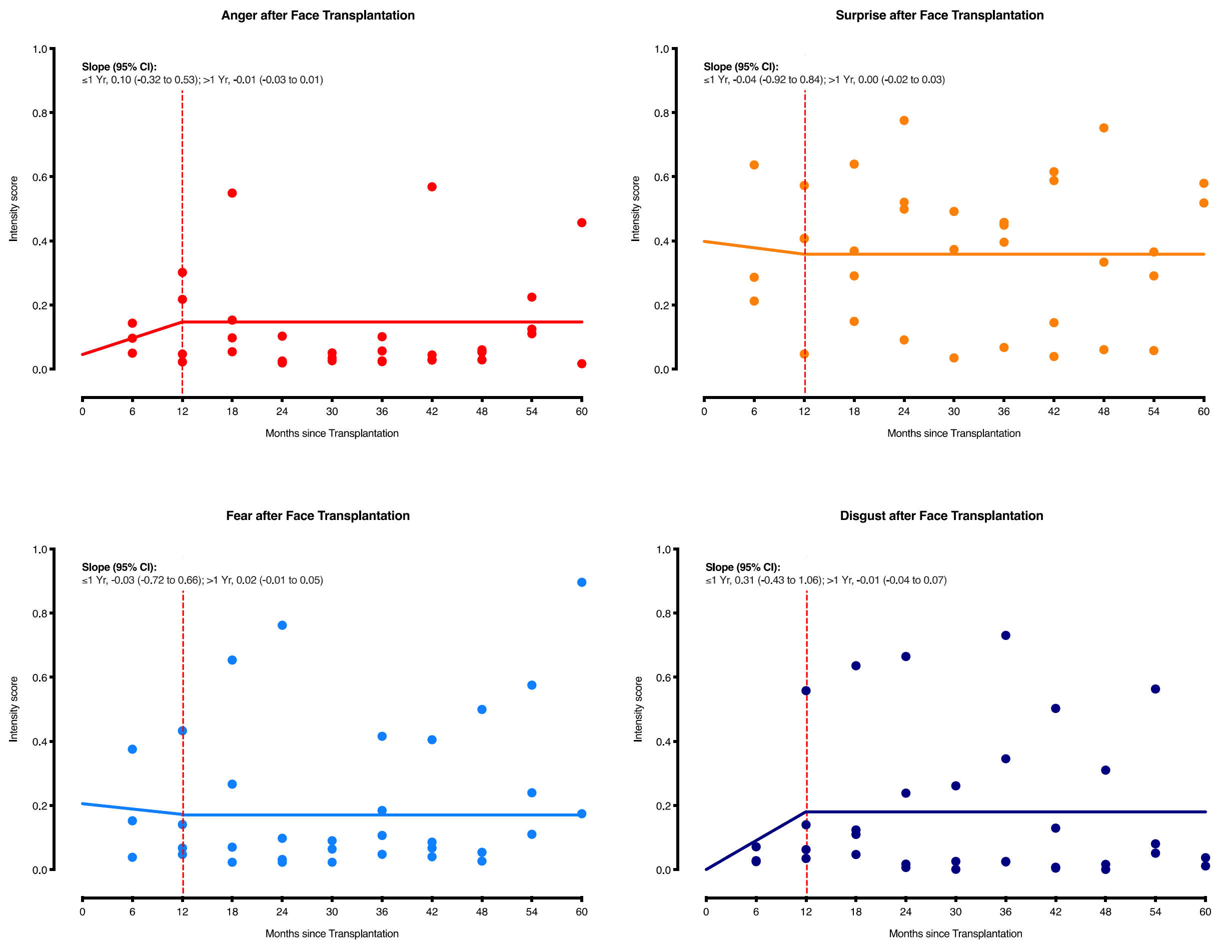

Longitudinal intensity score values of emotional expressions of anger, surprise, fear and disgust during indirect evaluation for patients with face transplants according to piece-wise linear regression model. None of these emotions were found to have intensity score values with significant changes post-transplant.

© 2020 Dorante MI et al. *JAMA Network Open.*

eFigure 5. Long-term Comparison of Happiness

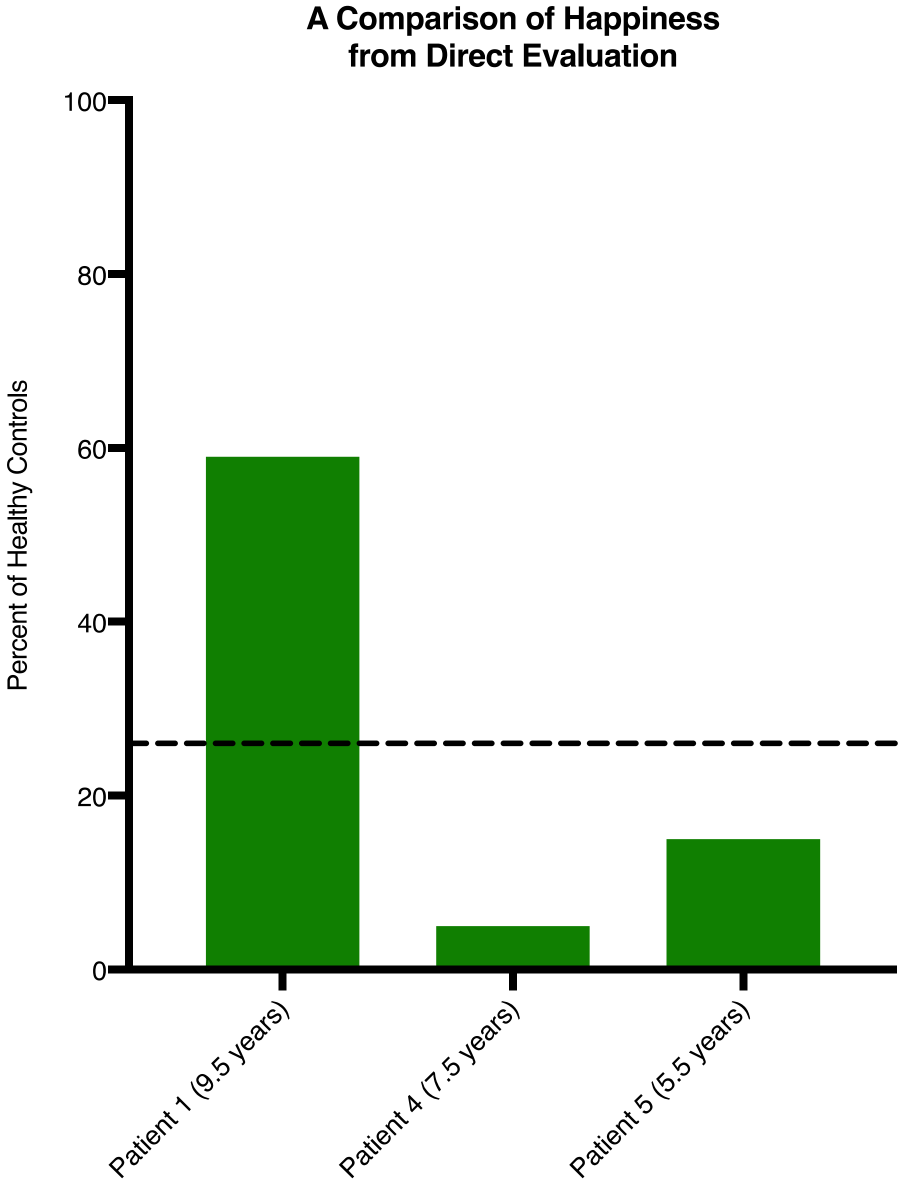

Maximum intensity score values of three patients with face transplants compared to the mean intensity score value of healthy controls for the emotional expression of happiness from direct evaluation. Based on limited cohort data, the happiness was shown to be restored to 26% of healthy subjects after face transplantation in the long-term.

© 2020 Dorante MI et al. *JAMA Network Open.*
